# Clinical Practice Update on the Evaluation, Diagnosis, and Management of Pancreatic Exocrine Insufficiency: Expert Review

**DOI:** 10.5152/tjg.2026.25679

**Published:** 2026-04-21

**Authors:** Orhan Sezgin, Dilek Oğuz, Müjde Soytürk, İsmail Hakkı Kalkan, Göksel Bengi, Özlem Gül

**Affiliations:** 1 Department of Gastroenterology, Mersin University Faculty of Medicine, Mersin, Türkiye; 2 Department of Gastroenterology, Kırıkkale University Faculty of Medicine, Kırıkkale, Türkiye; 3 Department of Gastroenterology, Dokuz Eylül University Faculty of Medicine, İzmir, Türkiye; 4 Department of Gastroenterology, TOBB University of Economics and Technology School of Medicine, Ankara, Türkiye; 5 Department of Gastroenterology, Lokman Hekim University School of Medicine, Ankara, Türkiye

**Keywords:** Exocrine pancreatic insufficiency, pancreatic enzyme replacement therapy, review

## Abstract

Pancreatic exocrine insufficiency (PEI) is characterized by inadequate synthesis, secretion, and/or activation of exocrine pancreatic secretions. This guideline has been developed to assist clinicians and researchers in understanding the definition, etiopathogenesis, epidemiology, diagnosis, treatment, and follow-up of PEI based on the current literature. All statements within this review were developed through consensus among the authors based on a comprehensive literature review.

**Main Points**

- Pancreatic exocrine insufficiency (PEI) can result from both pancreatic and extrapancreatic causes, with chronic pancreatitis being the most common cause in adults.
- Pancreatic exocrine insufficiency can present with symptoms common to other gastrointestinal disorders, such as bloating, nausea, abdominal pain, and diarrhea, in the early stages, whereas steatorrhea and weight loss can develop in the later stages.
- Patients with symptoms suggestive of PEI and/or underlying pancreatic disease can be diagnosed using a combination of data from patient-reported questionnaires, pancreatic imaging methods, and pancreatic function tests.
- All patients diagnosed with PEI should receive pancreatic enzyme replacement therapy (PERT). In adults, the recommended starting dose of PERT is 40 000-50 000 lipase units with main meals and 20 000-25 000 units with snacks.

## Introduction

Pancreatic exocrine insufficiency (PEI) is characterized by the inability to synthesize, secrete, and/or activate exocrine pancreatic secretions, including pancreatic enzymes and bicarbonate, at sufficient levels. Exocrine insufficiency leads to malnutrition because of maldigestion and malabsorption of nutrients. Consequently, symptoms such as steatorrhea and weight loss can significantly decrease the quality of life (QoL) and may even lead to life-threatening complications. The absence of overt symptoms, particularly in the early stages, coupled with challenges in accessing diagnostic tests, often results in PEI being overlooked and inadequately treated in clinical practice.

This review provides an evidence-based synthesis of the current understanding of PEI. It includes the definition, pathophysiological basis, epidemiology, etiology, diagnostic modalities, therapeutic management, and follow-up protocols associated with this condition.

## Methodology

This review was developed using an evidence-based methodology based on a systematic literature review addressing key aspects of PEI, including definition, etiology, diagnosis, treatment, prognosis, and follow-up.

In the initial phase, a working group of 6 experts was established to conduct the review. This group convened in May 2023 for an in-person meeting to delineate the objectives, main topics, and methodology for preparation of the review. During this meeting, it was determined that using the patient–intervention–comparator–outcome (PICO) methodology would be appropriate for the preparation of the review. A total of 12 questions were identified, and tasks were allocated to address these.

After a consensus meeting, a systematic literature review was initiated. Each expert was responsible for conducting a structured and question-specific search strategy within their respective domains of inquiry. Searches were performed in major English-language biomedical databases, including PubMed/MEDLINE, Cochrane Library, and Embase. The review process prioritized high-quality evidence, with an initial focus on randomized controlled trials and meta-analyses. Literature retrieval followed an iterative approach; systematic reviews and meta-analyses were examined first. In the absence of such data, the search was progressively broadened to include randomized controlled trials, cohort studies, and, when necessary, case reports in accordance with the established hierarchy of evidence.

During the final in-person meeting convened in May 2025, publications identified for each predefined question along with the corresponding extracted data were systematically reviewed and critically appraised. Based on a collective evaluation of the available evidence, consensus-driven recommendations were formulated for all the topics under consideration. Following this meeting, all sections were integrated and harmonized to finalize the expert review.

## Results

### Definition

Question 1: What is PEI?

Statement 1: PEI is the condition in which exocrine pancreatic secretions (pancreatic enzymes and bicarbonate) are not synthesized, secreted, and/or activated at adequate levels.

PEI is characterized by inadequate secretion of pancreatic enzymes and/or sodium bicarbonate or a reduction in pancreatic enzyme activity below the necessary threshold, leading to impaired nutrient digestion, particularly of fats.^1^ PEI may manifest through symptoms such as steatorrhea, weight loss, and certain biochemical abnormalities, as well as a decrease in QoL and the development of life-threatening complications. Nevertheless, it can remain underdiagnosed and insufficiently treated in routine clinical practice.^2^

PEI usually occurs when digestive enzymes are not synthesized because of damage to the functional pancreatic parenchyma or impaired vagal innervation that stimulates enzyme secretion. Additionally, PEI may result from the inability of synthesized enzymes to become activated in the duodenum, increased enzyme inactivation, or failure of enzymes to come into contact with nutrients in the duodenum.^3^^,^^4^

Pancreatic enzymes play a crucial role in the process of normal digestion, and deficiency in their exocrine functions can lead to malnutrition owing to impaired digestion and absorption of nutrients.

The stages at which deficiencies can develop are summarized as follows:

- Pancreatic secretion synthesis: Damage to the pancreatic parenchyma results in decreased production and secretion of bicarbonate from the pancreatic ducts and pancreatic enzymes from acinar cells. This functional loss can occur in diseases such as chronic pancreatitis (CP), cystic fibrosis (CF), pancreatic cancer, acute necrotizing pancreatitis, or pancreatic resection.
- Pancreatic stimulation: Diseases that reduce the release of cholecystokinin (CCK) from the duodenal mucosa, such as previous pancreatic/gastrointestinal (GI) surgery or celiac disease, lead to inadequate activation of pancreatic secretions. Additionally, somatostatin and its analogs, which are physiological pancreatic secretion inhibitors, can cause PEI.^2^
- Pancreatic secretion transport: Obstruction of the pancreatic duct prevents pancreatic secretions from reaching the intestinal lumen, thereby impairing digestion. This occurs in various pancreatic tumors or diseases such as CF, which leads to increased viscosity of secretions.
- Synchronization of GI secretions: Disruption of synchronization necessary for the interaction between biliopancreatic secretions and food can lead to digestive problems. Such problems typically arise from anatomical changes following pancreatobiliary or GI surgery.^1^

Question 2: In which diseases and conditions is PEI observed?

Statement 2:

- PEI can occur due to both pancreatic and extrapancreatic causes.
- The main pancreatic causes are CP, acute pancreatitis (AP), pancreatic cancer, CF, and pancreatic surgery.
- Extrapancreatic causes include diabetes mellitus (DM), inflammatory bowel disease (IBD), celiac disease, human immunodeficiency virus (HIV) infection, GI surgery, aging, and use of somatostatin and its analogs.
- The most common cause in adults is CP and in children it is CF.

Because PEI has various etiologies, its true prevalence remains unknown. The most common cause in adults is CP, whereas in children, it is CF.^5^^-^^9^ The pancreatic and extrapancreatic conditions that cause PEI are listed in Table 1.

PEI is one of the major complications of CP and should be considered in all patients diagnosed with CP.^10^ PEI in CP results from progressive destruction of the pancreatic parenchyma, leading to loss of function and a subsequent reduction in pancreatic secretions. Steatorrhea manifests at an advanced stage of severe PEI, typically occurring after 90%-95% of pancreatic parenchymal function has been lost.^11^ Moreover, steatorrhea has been documented in patients exhibiting secretory function exceeding 10%, indicating that individuals with less severe conditions may have delayed diagnosis.^12^ The probability of developing PEI within 10-12 years of CP diagnosis has been reported to be 60%-90%.^13^ Furthermore, the probability of PEI occurring in mild pancreatitis is 30%, whereas this rate can increase to 85% in severe pancreatitis.^14^ Alcohol and hereditary etiology, pancreatic duct obstruction, presence of calcification, smoking history, and disease duration are factors contributing to the development of PEI in CP.^2^^,7^ Studies have also shown that mortality increases in patients with CP who develop PEI.^15^ Factors such as alcohol consumption, hereditary etiology, obstruction of the pancreatic duct, calcification, smoking history, and disease duration contribute to the development of PEI in patients with CP.^2^^,7^

PEI may occur as a consequence of substantial parenchymal loss following an AP attack. A recent meta-analysis reported an average incidence rate of 62% for the development of PEI after AP, which increased to 66% in cases of severe AP. In a long-term follow-up, PEI persisted in 35% of these patients.^16^ Factors contributing to the development of PEI include the recurrence and severity of AP, the extent of pancreatic necrosis, and alcohol use. Therefore, measurement of exocrine pancreatic function after severe AP attacks is recommended.^17^

The incidence of PEI in pancreatic cancer, particularly in tumors located in the pancreatic head, ranges from 46% to 100%. In cases of inoperable tumors, exocrine function has been reported to decline by approximately 10% per month. Therefore, it should be kept in mind that PEI may occur later in the course of the disease, even if it is not evident at the time of diagnosis.^18^ Obstruction of the main pancreatic duct by the tumor and parenchymal atrophy play an important role in the development of PEI associated with pancreatic cancer. Furthermore, biliopancreatic obstruction results in decreased bicarbonate secretion, leading to an acidic intraluminal pH in the upper small intestine. This leads to the inactivation of secreted pancreatic enzymes or a reduction in their activity.^19^ Weight loss in patients with pancreatic cancer may be exacerbated by PEI-related malabsorption. In the largest prospective study on this topic, Partelli et al^20^ demonstrated that fecal elastase (FE) levels are an independent risk factor for survival, particularly in patients with advanced pancreatic cancer. In this study, patients with FE-1 levels >200 µg/g had a median survival of 11 months, whereas those with FE-1 levels ≤200 µg/g had a median survival of 7 months.

Postoperative anatomical and hormonal alterations after pancreatic surgery can result in PEI and maldigestion. A review of 9 observational cohort studies (n = 693) reported a mean preoperative prevalence of PEI of 44% in cases of pancreatic or periampullary tumors prior to pancreatoduodenectomy, 20% before distal pancreatectomy, 63% before total pancreatectomy, and between 25% and 50% in locally advanced pancreatic cancer. The severity of PEI is influenced by factors such as preoperative exocrine function, primary diagnosis, the type of surgical resection, and the volume of removed tissue.^21^ The study by Yuasa et al^22^ indicated that the incidence of PEI is higher in patients who undergo pancreaticoduodenectomy compared to those who undergo distal pancreatectomy. Pancreatic exocrine insufficiency after pancreatic surgery is associated with multiple mechanisms. This is particularly evident in the classic Whipple procedure, where removal of the duodenum results in decreased CCK release, disruption of the digestive synchrony between the stomach and pancreas, and reduction in functional pancreatic tissue. Furthermore, impaired fundal motility may impair gastric emptying and reduce the efficacy of pancreatic enzymes. Reconstruction technique represents a critical determinant in the pathogenesis and progression of postoperative PEI. Comparative investigations examining pancreaticogastrostomy (PG) and pancreaticojejunostomy (PJ) anastomotic approaches have demonstrated that PG may predispose patients to pancreatic enzyme inactivation because of exposure to low gastric pH, thereby increasing the incidence and severity of PEI. In a study conducted by Jang et al, all individuals undergoing PG exhibited severe PEI, whereas those reconstructed with PJ predominantly manifested milder insufficiency.^23^

Perez Aisa et al^24^ found that 38% of patients who underwent partial or total gastrectomy had PEI. In a recent study, exocrine pancreatic function was assessed through a combination of symptoms, FE-1 measurements, and therapeutic response to pancreatic enzyme replacement therapy (PERT) after 52 months of follow-up in 188 patients who underwent Roux-en-Y gastric bypass surgery for obesity; 31% developed PEI.^25^

CF accounts for approximately 85% of PEI cases manifesting in early childhood. Affected children experience a progressive decline in pancreatic exocrine function despite normal enzymatic activity at birth.^18^ In addition to CF, PEI may also arise in association with rare genetic syndromes, including Shwachman–Diamond, Johansson–Blizzard, and Pearson syndromes.^9^ Congenital forms of PEI unrelated to CF are exceedingly uncommon, and exocrine dysfunction typically constitutes a component of a broader multisystem disorder.^26^

Owing to the intricate anatomical and physiological interconnection between the exocrine and endocrine components of the pancreas, pathologies within the endocrine tissue can precipitate disorders in exocrine function and vice versa. The reduction in exocrine function in DM has been attributed to several factors, including an imbalance between stimulatory and inhibitory islet hormones, pancreatic atrophy or fibrosis, autonomic neuropathy, decreased release of GI mediators, and autoimmunity.^27^ PEI observed in DM is usually mild to moderate, and steatorrhea rarely occurs.^9^ PEI may develop due to diabetic exocrine pancreatic pathology, and underlying CP may be overlooked in patients diagnosed with DM. A retrospective study evaluating 1868 patients with newly diagnosed diabetes reported that 9.2% of the cases had pancreatogenic diabetes, with half of these cases being managed as type 2 DM.^28^ A meta-analysis determined the prevalence of PEI in individuals with type 1 and type 2 DM to be 39% and 28%, respectively.^29^ A subsequent meta-analysis conducted in 2022, which included 12 studies, reported the prevalence of PEI in patients with type 2 DM to be 22%, with 8% of these cases progressing to severe pancreatic insufficiency.^30^ Furthermore, a comprehensive study involving patients with diabetes identified a significant association between PEI and factors such as early onset and long-term DM, insulin usage, and low body mass index (BMI).^31^ Additionally, inadequate glycemic control, diminished residual beta cell function, and high BMI have been associated with reduced FE levels.^32^^,^^33^

In patients with IBD, the prevalence of PEI ranges from 18% to 80% in FE-based assessments.^34^^,^^35^ It has been demonstrated that transient PEI can occur during periods of IBD activation, in addition to its association with type 2 autoimmune pancreatitis (AIP), and that PEI tends to regress when the disease enters remission.^36^^,^^37^ This finding suggests that PEI may manifest as an extraintestinal complication of IBD. In Crohn’s disease, the prevalence of PEI was found to be between 14% and 30% in FE-based assessments.^35^^,^^36^ This decline in pancreatic function has been linked to IBD activity, disease location, and the affected intestinal area. The occurrence of PEI in Crohn’s disease is attributed to the presence of pancreatic autoantibodies, duodenal reflux, and reduced secretion of secretory hormones.^27^ Among patients with ulcerative colitis, 22% exhibited PEI (FE-1 ≤200 µg/g) and 9% had severe PEI (FE-1 ≤100 µg/g).^36^ It should be noted that the FE test may yield inaccurate results due to the presence of diarrhea and diluted stool in IBD.

Although PEI occurring in celiac disease is thought to be multifactorial, the most prevalent theory is the impaired release of pancreatic stimulating hormones from the atrophied proximal small intestine.^38^ Diarrhea may persist in 17%-61% of patients with celiac disease despite treatment, and PEI should be considered in this patient group.^39^^,^^40^ In patients with celiac disease with persistent diarrhea, PEI rates on a gluten-free diet have been found to range from 12% (based on pancreatic tests or response to PERT) to 18% (based on the presence of steatorrhea or response to PERT).^39^^,^^41^ In a meta-analysis published in 2021 that included a total of 6 studies and 446 patients, the incidence of PEI was found to be significantly higher in newly diagnosed patients (26.2%) compared to those on diet therapy (8%). Additionally, PEI was more likely to occur in patients with persistent symptoms in the diet group compared to those who were asymptomatic.^42^

In Zollinger–Ellison syndrome, PEI can occur in 5%-10% of cases because of inactivation of pancreatic enzymes by acid hypersecretion and decreased pH.^3^^,^^4^

Nonalcoholic fatty pancreatic disease (pancreatic steatosis) can lead to damage to the pancreatic tissue associated with chronic inflammation, leading to DM, pancreatic adenocarcinoma, and/or PEI. However, information on this topic is not yet clear.^43^^-^^45^

The geriatric population is prone to malabsorption and nutritional deficiencies due to changes in GI function and comorbidities associated with the physiological aging process. PEI is often underdiagnosed in this age group but significantly affects QoL. Aging leads to a decline in both exocrine and endocrine functions of the pancreas to varying degrees. Postmortem histological examinations demonstrated atrophy, fatty infiltration, and fibrosis in the pancreatic tissue with age. Consequently, pancreatic enzyme production and secretion decrease, leading to impaired fat digestion and absorption.^46^^,^^47^ The volume, structure, and secretory capacity of the pancreas change with age, particularly from the fifth decade of life onward. Therefore, after the age of 70 years, mild PEI is observed in 5% of cases, and severe PEI is observed in 10%.^45^

Among the infectious diseases, PEI is most commonly observed in HIV-positive patients. A recent prospective study found moderate reductions in FE in 32% of patients and severe reductions in FE in 20% of patients receiving antiretroviral therapy.^48^

Pancreatic damage caused by immune checkpoint inhibitors is a rare immunotoxicity, and limited data are available regarding its treatment and long-term outcomes. An analysis of 25 retrospective studies found a 10.5% probability of developing PEI due to these medications.^49^

A cross-sectional study of smokers without known pancreatic disease found moderate and severe FE reductions in 18% and 10% of patients, respectively, compared with controls.^50^

Question 3: What are the clinical manifestations of PEI?

Statement 3: The manifestations of PEI may include diarrhea, steatorrhea, abdominal distention, gas, bloating, abdominal pain, and/or weight loss.

In its initial stages, PEI may manifest with symptoms that are commonly associated with other GI disorders, including bloating, nausea, abdominal pain, and diarrhea. In the advanced stages, steatorrhea and weight loss occur as pancreatic lipase secretion decreases to less than 10% of normal levels.^51^

Protein deficiencies, along with deficits in trace elements such as magnesium and zinc and fat-soluble vitamins (A, D, E, and K), may arise as a consequence of PEI.^52^ Even in patients where PEI is mild or moderate, the impaired absorption of fat-soluble vitamins can lead to conditions such as osteomalacia, osteoporosis, and renal failure. Consequently, the early and precise diagnosis of PEI is essential. It is imperative to periodically assess bone mineral density in these patients, as the risk of nontraumatic fractures due to osteoporosis and osteopenia associated with PEI is increased.^7^

Moreover, a study by Shintakuya et al^53^ showed that the majority of patients with PEI developed sarcopenia despite being overweight. Furthermore, PEI is an independent risk factor for cardiovascular events, distinct from known risk factors (hypertension, diabetes mellitus, smoking, and obesity).^54^ Consequently, PEI causes increased morbidity and mortality rates.^55^ A decrease in QoL is another significant problem associated with PEI. Therefore, PEI should be considered in patients with risk factors, even in the presence of nonspecific symptoms.

### Diagnosis

Question 4: How is PEI diagnosed?

Statement 4:

- In patients with symptoms suggestive of PEI and/or underlying pancreatic disease, the diagnosis is based on a combination of nutritional assessment, standardized patient-reported questionnaires, pancreatic imaging methods, and pancreatic function tests. However, no definitive diagnostic criteria for PEI have been established.
- In clinical practice, the use of direct invasive tests for the diagnosis of PEI is not recommended.
- Fecal elastase-1 or ^13^C-mixed triglyceride (^13^C-MTG) breath tests can be used as noninvasive diagnostic tests. The FE-1 test is recommended because of its accessibility and ease of application.
- Imaging methods have no role in the diagnosis of PEI but are valuable in investigating its etiology.
  - In symptomatic patients with confirmed pancreatic disease, if nutritional assessment and pancreatic function tests do not help in the diagnosis, evaluation of clinical response to empirical PERT may aid in the diagnosis of PEI.

The clinical presentation of PEI is a combination of symptoms of malabsorption/maldigestion and signs of malnutrition. Deficiencies in vitamins and trace elements, particularly fat-soluble vitamins, as well as osteoporosis, bone fractures, sarcopenia, and a decreased QoL may occur.^56^ Patient questionnaires, particularly the Pancreatic Exocrine Insufficiency Questionnaire (PEI-Q), have also been introduced to assess PEI. A recently published Turkish validation study of the PEI-Q demonstrated that it had a sensitivity of 73% and a specificity of 90% in diagnosing PEI.^57^

The differential diagnosis for PEI is broad, and multiple conditions may coexist in a single patient, making diagnosis challenging. Integrating symptoms, clinical signs, and patient-reported questionnaires with pancreatic function tests enhances the reliability of the differential diagnosis. Further assessment for PEI is not advised, particularly in patients with pancreatic head canceror those who have undergone total pancreatectomy or pancreaticoduodenectomy.^58^^-^^62^

Pancreatic function tests are divided into direct and indirect methods. Direct pancreatic function tests assess the volume and composition of pancreatic secretions, including electrolytes and enzymes, following stimulation with secretin or cholecystokinin (CCK). Early techniques used a double-lumen gastric–duodenal tube (1940), but this invasive and radiology-dependent method was replaced by endoscopic collection of duodenal fluid.^63^ In a study by Raimondo et al^64^, endoscopic testing in 412 patients with suspected pancreatic disease showed reduced lipolytic activity in CP compared to controls, whereas bicarbonate and trypsin levels did not differ significantly. Lipolytic activity better distinguished CP from non-pancreatic disease. To avoid prolonged intubation, Ochi et al^65^ evaluated a direct method via ERCP, in which pancreatic fluid was collected after CCK and secretin stimulation. This approach demonstrated high sensitivity (87.5%-100%) and specificity (66.7%-90.9%) compared with the duodenal secretin test. Currently, direct tests are performed endoscopically in specialized centers and are mainly used for early diagnosis of CP rather than for diagnosing PEI.^66^ Although considered the standard for assessing exocrine secretion, direct tests are not recommended in routine clinical practice because they are invasive and time-consuming and require anesthesia.^63^ Furthermore, although these tests quantify stimulated secretion, they do not assess whether enzyme output is sufficient for digestion. The European guidelines also emphasize their limited utility in diagnosing PEI.^62^

Among the various indirect assessments, the FE-1 test is the most preferred test for evaluating pancreatic function due to its simplicity, noninvasive nature, and relative cost-effectiveness. Because FE is the digestive enzyme most likely to be excreted in the stool during intestinal transit, it is an indirect measure of pancreatic digestive enzyme production capacity.^66^ In clinical practice, FE-1 levels <200 µg/g are considered mild PEI, and levels <100 µg/g are considered severe PEI.^67^ A recent real-world study has shown that FE-1 levels <100 µg/g are more sensitive compared to those <200 µg/g in predicting steatorrhea. Furthermore, FE-1 monitoring has been shown to predict the progression of PEI.^68^ In a study by Leodolter et al^69^, the diagnostic accuracy of FE-1 and the pancreolauryl test (PLT) in 40 patients with radiologically diagnosed CP was compared. The sensitivity of PLT for diagnosing PEI across all severities was 82% (27/33), whereas for FE-1 it was 50% (16/33). In patients with severe PEI, PLT was abnormal in 100% (13/13) of patients, whereas FE-1 was abnormal in 85% (11/13).

In a meta-analysis of 14 studies using the secretin stimulation test as the gold standard (428 cases with PEI and 673 controls), the FE-1 test demonstrated a sensitivity of 77% and a specificity of 88% for diagnosing PEI. Studies using fecal fat determination as the gold standard have reported even higher values of sensitivity and specificity.^70^

The FE-1 test has low sensitivity in diagnosing mild PEI.^69^ The test is more accurate when analyzed in formed stool samples. Clinicians should be aware that semi-formed and watery stool samples may be diluted, which can lead to inaccurate results.^66^

Another indirect test used in the diagnosis of PEI is the ^13^CMTG breath test. This test measures ^13^C-labeled CO_2_, a breakdown product of digested triglycerides, and its amount is related to the current lipase activity; in other words, the test indirectly measures pancreatic function.^71^ Although various lipids have been utilized in breath tests, the modified version of the original test conducted by Vantrappen et al^72^, which employs mixed triglycerides, is currently the most widely utilized method. Studies using direct secretin ± cerulein tests as the gold standard have achieved high sensitivity (90%-100%) and specificity (80%-90%) for the diagnosis of severe PEI.^73^^,^^74^ A recent meta-analysis examined 6 studies (with a test meal containing at least a 10 g fat load) using fecal fat determination, FE-1, or direct tests as references and showed that the ^13^CMTG breath test had a sensitivity of 84% and a specificity of 87% for the diagnosis of PEI.^75^ However, detection of ^13^CO_2_ in breath is not only associated with lipase deficiency; other causes of malabsorption (celiac disease, short bowel syndrome, and gastric resection) may also yield a positive test result. Therefore, its specificity in the differential diagnosis of chronic diarrhea is low.^76^^,^^77^

Despite being recognized as the gold standard for PERT indication and treatment monitoring by both the United States Food and Drug Administration and the European Medicines Agency, the fecal fat absorption test (coefficient of fat absorption [CFA]) is challenging to implement in clinical practice due to its lack of specificity for PEI, low patient compliance, and limited acceptability.^71^

In cases where PEI is suspected, abdominal ultrasonography (USG) and cross-sectional imaging methods are used to identify underlying pancreatic disease.^66^ Calcification and main pancreatic duct dilatation due to CP can be visualized through USG and computerized tomography (CT). Although pancreatic calcification, atrophy, and duct dilatation are late or severe findings of PEI, a study of 109 patients with PEI found the described morphological findings in only 47% of those with severe PEI.^78^ Another investigation demonstrated that ductal dilatation observed during ERCP exhibited a stronger correlation with PEI compared to calcification.^79^ Furthermore, other studies have shown that non-severe morphological changes of the pancreas detected by CT, magnetic resonance imaging (MRI), or magnetic resonance cholangiopancreatography (MRCP) do not exhibit a correlation with PEI.^80^^-^^83^

In secretin-enhanced MRCP (s-MRCP), secretin, a synthetic agent administered intravenously (1 mL/10 kg body weight), improves visualization of the pancreatic duct by increasing its diameter. Indications for this technique include the detection and characterization of pancreatic duct anomalies and stenoses, assessment of pancreatic duct integrity, and characterization of a potential relationship between the pancreatic duct and pseudocysts/pancreatic fistulas. Another advantage is that, unlike other cross-sectional imaging methods, it can assess pancreatic function quantitatively in addition to its qualitative assessment. However, the difficulty in obtaining secretin in most countries is a major drawback of this test.^84^

Endoscopic ultrasonography (EUS) demonstrates early parenchymal changes in non-calcific CP with 84% sensitivity and 80%-100% specificity. However, in assessing histological changes (fibrosis score), sensitivity increases as the number of morphological changes detected by EUS increases. In the study by Trikudanathan et al, CP was histopathologically detected in 55% of cases with a normal Rosemont.^83^^,^^85^^,^^86^

In a study investigating the effectiveness of EUS in demonstrating pancreatic function using s-MRCP as the gold standard, the positive predictive value of EUS for PEI was found to be only 50%.^87^ Although new imaging modalities such as elastography, EUS, and pancreas/spleen T1 signal intensity are promising, comparative studies are needed to compare the gold standard reference values.^81^^,^^88^

Diagnosing PEI can pose diagnostic challenges despite the combined evaluation of symptoms, signs of malnutrition, and indirect pancreatic function tests. Therefore, among patients with established pancreatic disease, improvement of symptoms and malnutrition with empiric PERT may support the diagnosis of PEI. Although evidence supporting this approach is lacking, this recommendation is included as an expert opinion in 2 separate (Europe and UK) guidelines.^6^^,^^62^

Table 2 shows a stepwise diagnostic approach and intervention pathway for PEI.

### Treatment

Question 5: In which cases should PEI be treated?

Statement 5: Every patient diagnosed with PEI should be treated.

PERT forms the basis of PEI treatment. Accurately determining the appropriate enzyme dosage is crucial for ensuring effective therapeutic outcomes. The goals of this treatment are to restore normal digestive function, alleviate PEI-related symptoms, improve QoL, and reduce malnutrition-related morbidity and mortality. Meta-analyses have shown that PERT enhances protein and fat absorption compared to placebo.^89^^,^^90^

Because lipase requirements may be higher in advanced-stage CP or CF, “dosing per kilogram” is preferred. The recommended initial dose is 40 000-50 000 units of lipase to be taken with main meals, whereas half of this dosage should be administered with snacks.^91^^-^^93^

Although PERT has been shown to improve survival in patients with pancreatic ductal adenocarcinoma, its use remains relatively low.^94^ A retrospective study by Domínguez-Muñoz et al showed that the addition of PERT to nutritional therapy and palliative care in unresectable pancreatic cancers had a positive effect on median survival.^95^ PERT improves QoL and assists in managing weight loss in patients with advanced pancreatic cancer.

Pancreatic surgery plays an important role in the treatment of malignant and benign lesions in both the pancreatic and periampullary regions. The most performed surgical procedures include pancreaticoduodenectomy (Whipple operation), pylorus-preserving pancreaticoduodenectomy, distal pancreatectomy, and total pancreatectomy.^96^ PEI due to pancreatic resection is a clinical condition that can be overlooked but can negatively impact QoL and survival. It is particularly common after pancreaticoduodenectomy. Accurate diagnosis and effective treatment of PEI are essential components of postoperative care. PERT should be applied on an individualized basis in these patients.^58,97-^^99^

In both type 1 and type 2 DM, PEI is associated with autoimmune destruction, which is subsequently followed by steatosis and fibrosis. PEI is notably prevalent, particularly in type 3c DM, which is classified as pancreatogenic DM. However, recent studies have revealed that PEI may be more prevalent than it was previously recognized, manifesting at subclinical or clinical levels in patients with type 1 and 2 DM. In this context, PERT stands out as a treatment modality offering potential benefits not only in alleviating malabsorption symptoms but also in improving metabolic regulation. PERT should be administered in accordance with established standard principles. However, dosage should be dynamically individualized based on the patient’s symptomatic profile, fecal elastase level, anthropometric measurements, and fecal fat content. Treatment response should be assessed within the first few weeks. Long-term follow-up should include clinical evaluation with parameters such as fat-soluble vitamin levels, serum albumin and prealbumin levels, complete blood count, and HbA1c.^100^^,^^101^ Several randomized controlled trials in the literature have examined the effects of PERT on glycemic parameters and have reported significant improvements, particularly in postprandial hyperglycemia and glycemic fluctuations.^102^ Therefore, PERT should be evaluated from a broader perspective within multidisciplinary diabetes management strategies.

PERT should be considered in selected patients with celiac disease to alleviate GI symptoms and control malabsorptive symptoms. Recent randomized controlled trials have shown that PERT may be particularly beneficial in patients with celiac disease whose symptoms persist despite gluten-free diet treatment. A study by Evans et al^103^ reported that the addition of PERT to patients with celiac disease experiencing steatorrhea and weight loss despite a gluten-free diet resulted in a significant reduction in fecal fat and weight stabilization. Conversely, other randomized controlled trials indicated that PERT did not alleviate symptoms in patients with celiac disease who were unresponsive to dietary interventions.^104^

PERT is administered according to the general standard approach in celiac disease. Treatment response is generally observed within 1-2 weeks, after which the patient’s symptoms and nutritional parameters should be reevaluated. The need for long-term treatment should be tailored based on the persistence of PEI and the response of celiac disease to a gluten-free diet.

PERT in IBD is similar to the one in the management of PEI. Response to treatment should be assessed by monitoring symptoms, weight change, stool consistency, and nutritional biomarkers. Current evidence suggests that PERT may provide potential benefits in this patient group in terms of symptomatic relief and improved nutritional status. Nevertheless, this approach should be considered a supportive intervention for selected patients following a thorough diagnostic evaluation, rather than a standard treatment. Substantial evidence from large, randomized trials is necessary.^105^

The identification and management of PEI in geriatric populations are crucial for the prevention of age-associated conditions such as sarcopenia, weight loss, vitamin deficiencies, and overall frailty. In this context, PERT emerges as a significant therapeutic option, particularly for elderly individuals experiencing malnutrition and digestive symptoms. Although randomized controlled trials assessing the efficacy of PERT in geriatric populations remain limited, existing data suggest that this treatment effectively alleviates symptoms, prevents the onset of sarcopenia, facilitates recovery in patients with sarcopenia, and enhances nutritional parameters.^16^^,^^106^ PERT should be administered to geriatric patients in accordance with established guidelines. Dose titration should be guided by the patient’s clinical symptoms, whereas nutritional parameters—particularly serum vitamin D, albumin levels, and body weight—must be systematically monitored throughout the course of treatment. Treatment efficacy should be assessed within 2-4 weeks following initiation of therapy. Given the high prevalence and significant clinical impact of PEI among geriatric individuals, PERT represents a cornerstone in the management of malabsorption within this population. Current evidence indicates that PERT effectively alleviates GI symptoms, enhances nutritional status, and can be safely administered in elderly patients. Consequently, a more systematic evaluation of PERT indications and increased clinical awareness regarding its use in older adults are warranted.

Question 6: Which enzyme preparations should be used in the treatment of PEI?

Statement 6: Preparations containing a combination of amylase, lipase, and proteases should be used.

All preparations used in the treatment of PEI are derived from porcine pancreas. Patients should be informed of the porcine origin of PERT before starting the treatment. Plant-based enzyme preparations should not be used in the treatment of PEI as they lack standardized and quantifiable enzyme content. Enteric-coated formulations are resistant to gastric acid and dissolve in the small intestine at a pH >5.5, enabling lipase activation. Preparations are available in microspheric or minimicrospheric forms. Microsphere/minimicrosphere (1-2 mm) formulations offer the advantage of greater surface area and better mixing in the duodenum.^11^ Non-enteric-coated formulations offer the advantage of faster dissolution but are susceptible to degradation by gastric acid, requiring concomitant acid-suppressing therapy.^11^^,^^107^ These products typically contain lipase, amylase, and protease. The selection of treatment is based on lipase concentration.^89^

Microbial-derived pancreatic enzyme preparations, particularly formulations containing amylase, lipase, and protease derived from fungi such as *Aspergillus oryzae* and *Aspergillus niger*, offer an alternative for patients who do not wish to use porcine-derived enzymes. These preparations are generally vegan or vegetarian and may be preferred by individuals who reject porcine-derived products for religious or ethical reasons. Moreover, microbial enzymes produced through microencapsulation technology demonstrate resistance to gastric acid, thereby facilitating their effective release in the duodenum and enhancing bioavailability.^108^^,^^109^ Due to the paucity of randomized controlled trials and the lack of effective dose standardization, microbial preparations are reported to be insufficient as monotherapy, especially in patients with severe PEI. The digestive activity of microbial enzymes in the digestion of high-fat meals may be less effective compared to porcine pancreatic enzyme preparations, potentially limiting their clinical efficacy. It has been reported that microbial enzymes alone do not provide sufficient therapeutic effects, especially in patients with severe PEI. Therefore, the role of microbial enzymes in the treatment of PEI is limited to mild-to-moderate cases and requires an individualized, symptom-oriented approach.^11^ During their clinical use, fecal fat excretion, weight control, and monitoring of GI symptoms are important.

### Dosing Principles

Question 7: What should be the dose of PERT?

Statement 7: For adult patients, the starting PERT dose should be 40 000-50 000 lipase units with main meals and 20 000-25 000 units with snacks. Although there is no established upper dose limit for treatment, it is recommended not to exceed 10 000 lipase units/kg/day.

PERT, which constitutes the cornerstone of therapeutic strategies for PEI, primarily aims to maintain adequate caloric and micronutrient intake. In addition, it seeks to mitigate GI symptoms that may arise as a consequence of steatorrhea or the consumption of meals with elevated fat content.^109^^,^^110^ A physiologically healthy pancreas is capable of secreting approximately 900 000 lipase units during an average meal. Notably, only 10% of this quantity, or approximately 90 000 lipase units, is required to prevent the onset of steatorrhea. Nevertheless, current PERT formulations exhibit lower bioavailability compared to the natural secretory capacity of the pancreas and may not fully suppress steatorrhea independently.^110^^,^^111^ Assuming that there is some residual pancreatic function in patients with PEI, a starting dose of 40 000-50 000 lipase units is recommended for main meals and half this amount for snacks. The dose should be individualized based on the fat content of the meal. It is recommended to increase the dosage for high-fat or voluminous meals and to decrease it for light, low-fat meals. Clinical experience and studies suggest that doses greater than 120 000 lipase units per meal are rarely necessary.^90^ Dosing beyond this level may yield minimal therapeutic gains and may increase side effects.^90^^,^^110^ Pancreatic enzymes should be administered at the commencement of a meal, with additional doses provided midway through or upon completion of the meal if clinically warranted. In certain patients, dosage reduction may be appropriate as clinical status and nutritional parameters stabilize. PERT is typically maintained as a lifelong intervention; therefore, treatment regimens should be individualized, as a fixed-dose approach is not recommended.^113^^,^^114^

Question 8: What are the major and minor side effects of PERT?

Statement 8: There are no major side effects associated with PERT.

PERT is considered a safe and efficacious intervention for the symptomatic management of PEI. Nevertheless, certain adverse events have been documented, particularly in association with prolonged or high-dose administration. The most commonly reported side effects are GI in nature and are typically mild and self-limiting. Transient abdominal distension, nausea, constipation, and diarrhea have been reported in patients receiving high-dose pancreatic enzyme preparations.^89^^,^^115^^,^^116^

These symptoms are typically dose-dependent, tend to manifest early in the course of therapy, and generally do not necessitate clinical intervention. Although infrequent, hypersensitivity reactions—including pruritus, urticaria, erythematous rash, muscle spasms, and blurred vision—have also been documented. These side effects may be related to immunological responses to the porcine protein structure of enzyme preparations. Pruritus is the most frequently reported immunological adverse reaction in the literature and may negatively impact patient compliance with treatment. If allergic symptoms occur, discontinuation of treatment and consideration of alternative approaches is recommended.^89^ Asymptomatic transaminase elevations have been reported in patients using PERT, emphasizing that this condition has limited clinical significance.

Although theoretical, the potential for zoonotic viral transmission through porcine-derived PERT preparations has been identified as a concern.^117^

High-dose PERT use may increase the risk of fibrosing colonopathy in patients with CF.^117^ This side effect has been associated with enzyme coatings containing methacrylic acid copolymers; however, it has also been observed in patients who are not undergoing PERT. Metabolic complications such as hyperuricosuria and associated nephrolithiasis have also been reported in children with CF in a dose-dependent manner.^118^

No clinically significant drug interactions have been identified to date with PERT preparations, suggesting a favorable profile in terms of pharmacokinetic and pharmacodynamic stability of the treatment.^11^

Question 9: What should be the nutritional recommendations for PEI?

Statement 9:

- PERT should be integrated with optimal nutritional strategies.
- If the patient’s symptoms do not improve, fiber restriction may be required.
- Fat restriction is not recommended; the use of medium-chain triglycerides (MCT) may be beneficial.
- Levels of fat-soluble vitamins and micronutrients should be monitored at regular intervals, and deficiencies should be replaced.

In addition to pharmacological intervention, an individualized, evidence-based nutritional approach should be implemented in the treatment of PEI. Patients with PEI typically exhibit increased energy requirements. Therefore, dietary modification is essential to compensate for nutrient losses resulting from malabsorption and to prevent the development of malnutrition. Energy demands should be estimated according to the individual’s age, sex, body weight, and comorbid conditions.^89^ Consuming smaller, more frequent meals throughout the day may increase the efficacy of PERT; however, the evidence supporting this recommendation remains limited due to the paucity of randomized controlled trials.^118^^-^^120^ Another important consideration is fiber intake. Soluble fiber, in particular, can interact with pancreatic lipase, preventing the enzyme’s access to fats.

This may impair lipid hydrolysis, thereby exacerbating malabsorptive manifestations such as steatorrhea, abdominal pain, bloating, and gas.^119^ In patients whose symptoms persist despite PERT, a moderated—rather than complete—restriction of soluble dietary fiber is advisable. Soluble fiber plays a pivotal role in modulating GI motility, sustaining eubiotic microbial communities, and promoting long-term GI homeostasis.^62^

Fat absorption is markedly compromised in patients with PEI. Therefore, modulation of dietary fat consumption constitutes a fundamental aspect of the nutritional management of this condition. Ensuring sufficient fat intake is essential not only for maintaining optimal energy.^109^^,^^110^ Patients have been shown to tolerate a normal-fat diet if adequate enzyme supplementation is provided through PERT. Total fat intake in individuals diagnosed with PEI should not be restricted; instead, malabsorption should be managed with adequate enzyme replacement. There are no adequate studies evaluating the impact of dietary fat restriction on clinical outcomes.

Patients with clinical symptoms despite adequate PERT should be evaluated for causes such as bile acid malabsorption.^89^^,^^11^^,^^62^ Screening for vitamin and mineral deficiencies (vitamins A, D, E, K, and B12, folate, magnesium, selenium, zinc, and iron) is necessary at the time of diagnosis and annually thereafter, depending on the patient’s clinical condition.^121^^,^^122^ Vitamin D levels and bone densitometry measurements should be monitored in patients with PEI, as vitamin D deficiency is associated with osteopathy and bone fractures, and treatment has been shown to reduce bone fracture rates.^123^^,^^126^

In certain clinical contexts—particularly in cases characterized by severe steatorrhea, weight loss, or insufficient energy intake, MCT-supplemented diets have been reported to be of temporary therapeutic utility. MCTs are absorbed directly into the portal circulation, bypassing the need for pancreatic lipase and bile acid-mediated emulsification. Because of these metabolic characteristics, MCTs may be preferred to increase energy support in patients with limited digestive ability. Nevertheless, as MCTs do not contain essential fatty acids, prolonged use may precipitate essential fatty acid deficiency. Accordingly, MCT supplementation should be confined to short-term, goal-directed application and individualized based on the patient’s specific nutritional requirements.^118^^,^^62^

Question 10: How should PERT be in special conditions that cause PEI?

Statement 10.1: In the treatment of CF patients with PEI, a general approach should be applied, and dosing should be done according to age and weight.

It is recommended that the total daily dose not exceed 10 000 lipase units/kg/day. This limit should be carefully monitored because of the risk of fibrosing colonopathy, a rare but serious complication.^91^^-^^93^

Statement 10.2: In the treatment of PEI secondary to pancreatic cancer, the general approach and standard-dose PERT should be applied.

PERT is particularly recommended in the presence of progressive weight loss and steatorrhea, defined as ≥7 g of fecal fat on a diet containing 100 g of fat per day.^94^^-^^97^ It has been reported that 25 000-50 000 U of lipase should be taken with a main meal for optimal lipid digestion.^97^

Although the use of PERT has been shown to extend survival in patients with pancreatic cancer, rates of PERT use are remain relatively low within this patient group.^98^ A retrospective study by Domínguez-Muñoz et al^97^ evaluated 76 patients with unresectable pancreatic cancer. The median survival of 45 patients who received PERT along with nutritional counseling and palliative care was significantly longer than that of 21 patients who received standard palliative care alone. A study by Woo et al^98^ also demonstrated the potential benefits of PERT in reducing the rate of weight loss in patients with unresectable pancreatic cancer. Furthermore, in a double-blind, placebo-controlled study of patients with unresectable pancreatic cancer, the mean weight loss in the PERT group (1.49%) was not significantly different from the placebo group (2.99%). However, this study found significant improvements in nutritional status in a subgroup of patients with tumors located in the pancreatic head.^99^

PEI developing secondary to pancreatic head tumors or pancreaticoduodenectomy is associated with pancreatic parenchymal loss and duodenal-pancreatic asynchrony. The starting dose of PERT is generally 40 000-50 000 U of lipase per main meals and 20 000-25 000 U at snacks. PERT improves QoL and mitigates weight loss, particularly in patients with advanced pancreatic malignancy. Combination therapy with proton pump inhibitors (PPIs) is recommended, particularly in cases of high gastric acidity.

Statement 10.3: In the treatment of PEI secondary to pancreatic surgery, the general approach and standard-dose PERT should be applied.

Pancreatic surgery plays an important role in the treatment of malignant and benign lesions in both the pancreatic and periampullary regions. The most commonly performed surgical procedures include pancreaticoduodenectomy (Whipple operation), pylorus-preserving pancreaticoduodenectomy, distal pancreatectomy, and total pancreatectomy. These interventions are particularly effective for conditions such as pancreatic head tumors, periampullary lesions, neuroendocrine tumors, and cystic neoplasms. PERT is one of the fundamental approaches in the management of PEI following pancreatic surgery. The goals of treatment are to relieve digestive disorders, prevent weight loss, and improve nutritional status. Current guidelines recommend the administration of lipase preparations containing 40 000-50 000 U of lipase at main meals and 20 000-25 000 U at snacks.^62^ In cases where the response is inadequate, enzyme activity may be enhanced by regulating gastric acidity with a PPI. Additionally, deficiencies of fat-soluble vitamins should be monitored and replaced if necessary.^23^^,^^127^

PEI associated with pancreatic resection is a clinical condition that can be overlooked but can negatively impact QoL and survival. PEI is particularly common after pancreaticoduodenectomy. Effective diagnosis and treatment of PEI should be an integral part of postoperative care, and PERT should be implemented on an individualized basis.

Statement 10.4: In the treatment of PEI secondary to DM, the general approach and standard-dose PERT should be applied.

In PEI associated with type 1 and 2 DM, PERT stands out as a treatment modality offering potential benefits not only in alleviating malabsorption symptoms but also in improving metabolic regulation. The recommended starting dose for PERT is generally 40 000-50 000 U of lipase with each main meal and 20 000-25 000 U of lipase with each snack. Nevertheless, the dosage should be tailored dynamically according to the patient’s symptomatic profile, FE-1 level, anthropometric measurements, and stool fat content. Treatment response should be frequently assessed within the first few weeks. Long-term follow-up should include clinical assessment, incorporating parameters such as fat-soluble vitamin levels, serum albumin and prealbumin levels, complete blood count, and HbA1c.^100^^,^^101^ Randomized controlled studies in the literature examined the effect of PERT on glycemic parameters and reported that it provided significant improvements, especially on postprandial hyperglycemia and glycemic fluctuations.^102^

Concomitant PEI can often be overlooked in diabetic patients, negatively impacting both QoL and metabolic control. Current evidence indicates that PERT not only alleviates symptoms but also exerts beneficial effects on glycemic control in certain diabetic patient populations, particularly those with type 3c diabetes.^126^ Therefore, PERT should be evaluated within a broader perspective within multidisciplinary diabetes management strategies.

Statement 10.5: In the treatment of PEI secondary to celiac disease, the general approach and standard-dose PERT should be applied.

PERT is recommended for patients with celiac disease with symptomatic PEI.^103^^,^^104^ An initial dose of 40 000-50 000 U of lipase is recommended with main meals and 20 000-25 000 U with snacks. The treatment dose is individualized based on symptoms and FE-1 levels. Typically, a response to treatment is observed within 1-2 weeks, at which point patient symptoms and nutritional parameters should be reevaluated. The necessity for prolonged treatment should be customized according to the persistence of PEI and the individual’s response to a gluten-free diet.

PEI secondary to celiac disease is a critical consideration in differential diagnosis, especially in patients whose symptoms persist despite adherence to a gluten-free diet. PERT is an effective supportive treatment for controlling GI symptoms and improving QoL in this patient group. Based on current data, it is recommended that PEI assessment in celiac disease be incorporated into routine clinical practice and that PERT be initiated when indicated.

Statement 10.6: In the treatment of PEI secondary to IBD, the general approach and standard-dose PERT should be applied.

The initial PERT dose is similar to that used in classical PEI management in patients with IBD, with 40 000-50 000 U of lipase administered with main meals and 20 000-25 000 U of lipase administered with snacks. Response to treatment should be assessed through the monitoring of symptoms, weight change, stool consistency, and nutritional biomarkers. Current evidence suggests that PERT may provide potential benefits in symptomatic relief and improved nutritional status in this patient group.^105^ However, this approach should be considered a supportive intervention in selected patients after careful diagnostic evaluation rather than as a standard treatment. Additional evidence derived from large-scale randomized trials remains essential.

Statement 10.7: In the treatment of PEI in geriatric population, the general approach and standard-dose PERT should be applied.

The PERT in geriatric patients is generally initiated with a dose of 40 000-50 000 U of lipase at main meals and 20 000-25 000 U of lipase at snacks. Dose titration should be guided by clinical symptoms, with ongoing monitoring of nutritional parameters—particularly serum vitamin D, albumin levels, and body weight—throughout the course of treatment. Response to treatment should be assessed within 2-4 weeks. Given the high prevalence and clinical implications of PEI among geriatric populations, PERT represents a key therapeutic intervention for managing malabsorption in this demographic. Current evidence indicates that PERT effectively alleviates symptoms, improves nutritional status, and can be administered safely.^16^^,^^106^ Therefore, a more systematic evaluation of the indications for PERT in older adults, along with increased clinical awareness, is warranted.

Statement 10.8: In the treatment of PEI in pregnancy, the general approach and standard-dose PERT should be applied.

Pregnancy is a dynamic process characterized by extensive physiological, hormonal, and GI changes. Clinical studies evaluating PEI have not included pregnant or lactating women. However, case reports on patients with CF and other diseases have not reported any adverse effects associated with PERT use during pregnancy.^127^^,^^128^ Increased energy and nutrient requirements during pregnancy may exacerbate the effects of PEI. Inadequate digestion and absorption can lead to complications such as maternal malnutrition, weight loss, and fetal growth retardation. Therefore, effective and safe use of PERT is crucial. Dosing of PERT during pregnancy should be administered according to recommended standard doses. Monitoring steatorrhea, weight gain, and nutritional status is important for dose adjustments. Furthermore, levels of fat-soluble vitamins should be regularly monitored and supplemented if necessary.^129^ PERT plays a critical role in the management of PEI during pregnancy and in safeguarding both maternal and fetal health. Current evidence regarding the safety of PERT during pregnancy remains limited. In clinical practice, the therapeutic efficacy and safety profile of PERT may be optimized through individualized dose titration and systematic clinical monitoring.

Question 11: How should the response to PERT be evaluated?

Statement 11: Both clinical and laboratory criteria should be concurrently assessed when monitoring treatment response and determining appropriate dose adjustments.

In the treatment of PEI, PERT is a fundamental approach for correcting malabsorption and improving nutritional status. However, clinical and laboratory criteria must be used together to assess the effectiveness of PERT, monitor treatment response, and make dose adjustments. Clinical assessment includes consideration of symptoms such as weight change, steatorrhea, abdominal pain, bloating, and gas, anthropometric measurements, and QoL. The PEI-Q can be used for this purpose.^57^ Laboratory assessments should include monitoring nutritional indicators such as serum albumin, prealbumin, and vitamin D levels.^89^ FE-1 is a useful diagnostic tool for the initial assessment and severity stratification of PEI; however, routine serial testing for disease follow-up or treatment response is not recommended. Coefficient of fat absorption, one of the objective assessment criteria, is a parameter that evaluates fat absorption by measuring the amount of fat in the stool. Meta-analyses show that PERT significantly increases CFA.^89^ The coefficient of nitrogen absorption (CNA) is another objective parameter that evaluates protein absorption. PERT has also been shown to have positive effects on CNA.^90^ Follow-up should be guided by clinical evaluation and monitoring of nutritional biomarkers, including serum albumin and vitamin D levels, which better reflected disease status and treatment.

Question 12: What are the recommended adjustments for the dosage in PERT follow-up?

Statement 12: Dose adjustment should be made in clinical situations where treatment is insufficient.

The success of PERT depends on systematic monitoring and dose adjustments throughout the treatment period. Treatment response should be monitored at regular 3- to 6-month intervals; however, the frequency of follow-up should be increased in patients with persistent clinical symptoms. If steatorrhea, abdominal distension, weight loss, or other signs of malnutrition persist, the patient’s adherence to therapy and correctness of enzyme use should be evaluated first. If the treatment is used correctly, the enzyme dosage should then be considered inadequate. In such cases, the first step is to adjust the enzyme dose according to the patient’s body weight and meal composition.^130^ If the response is still inadequate, the addition of a PPI should be considered. Alternatively, switching to different formulations (e.g., microspherical or minimicrosphere capsule structures) may be considered. Bioavailability, particle size, and bile stability of a preparation may influence individual differences in response to treatment.^131^ In treatment-resistant cases, other possible causes (e.g., bacterial overgrowth, secondary dysbiosis, and bile acid malabsorption) should be investigated, and additional treatment strategies should be formulated to address these specific conditions.^132^

A structured overview of the general principles of treatment, dose titration, follow-up, and response assessment in PEI is summarized in Table 3. Practical management considerations for special clinical situations are presented in Table 4.

### Summary

PEI is characterized by inadequate digestion of nutrients, particularly lipids, due to insufficient secretion of pancreatic enzymes and/or sodium bicarbonate, or when pancreatic enzyme activity falls below the necessary threshold level. It can occur due to both pancreatic and extrapancreatic causes. The most common causes in adults are CP and CF in children. PEI can present with symptoms common to other GI disorders, such as bloating, nausea, abdominal pain, and diarrhea, in the early stages, whereas steatorrhea and weight loss can develop in the later stages. Deficiencies of proteins, oligo-nutrients, and fat-soluble vitamins can occur because of PEI. In patients with symptoms suggestive of PEI and/or underlying pancreatic disease, it can be diagnosed by combining data from patient-reported questionnaires, pancreatic imaging methods, and pancreatic function tests. In symptomatic patients with proven pancreatic disease, if nutritional status and pancreatic function tests are not diagnostic, evaluation of the clinical response to empirical PERT may be helpful. Every patient diagnosed with PEI should be treated with PERT. Enteric-coated microsphere/minimicrosphere forms of PERT offer advantages due to their resistance to gastric acid and their extensive surface area. Determining the correct enzyme dose is essential for effective treatment. In adult patients, the starting PERT dose should be 40 000-50 000 lipase units with main meals and 20 000-25 000 units with snacks. It is recommended that the total daily dose should not exceed 10 000 lipase units/kg/day. In patients undergoing regular treatment monitoring at 3- to 6-month intervals, persistence of clinical symptoms should prompt consideration of dose escalation and the addition of a PPI.

## Figures and Tables

**Table 1.** Causes, Prevalence, and Associated Factors for the Development of PEI

Pancreatic Causes		
**Disease**	**Prevalence of PEI (%)**	**Factors Associated with Development of PEI**
Chronic pancreatitis^13^^,^^14^	30-90	Long disease duration Use of alcohol Widespread calcification Ductal obstruction
Acute pancreatitis^16^	Mild: 15-20 Severe: 30-40	Area of necrosis Use of alcohol
Pancreatic cancer^18^^,^^19^	20-60	Pancreatic head involvement Size Ductal obstruction Parenchymal atrophy
Pancreatic cancer surgery^21^	Pancreaticoduodenectomy: 80-90 Distal pancreatectomy: 20-50	Whipple operation Gastropancreatic anastomosis Decreased CCK release Disruption of digestive synchrony between the stomach and the pancreas Decrease in functional pancreatic tissue
Cystic fibrosis^18^	80-90	Class I, II, III, and VI CFTR mutations
Non-pancreatic causes		
Type 1 DM^29^^,^^30^	30-50	High-dose insulin requirement Inadequate glycemic control Early onset of diabetes
Type II DM^29^^,^^30^	20-30	Insulin requirement Inadequate glycemic control Long-term diabetes
Inflammatory bowel disease^34^^,^^35^	Ulcerative colitis: 10 Crohn’s disease: 4	Disease reactivation (only for temporary PEI) Disease duration Surgery Presence of pancreatic autoantibodies (autoimmunity) Duodenal reflux Decreased secretory hormone secretion
Celiac disease^41^^,^^42^^,^^43^	5-80	Untreated disease Impaired release of pancreatic stimulating hormones from atrophied proximal small intestine
Gastrointestinal surgery^24^	Total/subtotal gastrectomy: 40-80 Esophagectomy: 16	Extended bowel resection Vagal denervation
Bariatric surgery^25^	30-40	
HIV ^47^	10-50	Retroviral therapy
Age^45^	15-30	Age >80 years Atrophy, fatty infiltration, and fibrosis in pancreatic tissue
Smoking^48^	10-20	Use of alcohol
Use of somatostatin analogues^2^	20	

CCK, cholecystokinin; CFTR, cystic fibrosis transmembrane conductance regulator; DM, diabetes mellitus; HIV, human immunodeficiency virus; PEI, pancreatic exocrine insufficiency.

**Table 2.** Stepwise Diagnostic Approach and Intervention Pathway for PEI

**Scenario**	**Interpretation**	**Next Step for Diagnosis**
Symptoms + FE-1 <200 µg/g	Confirmed PEI	
Symptoms + FE-1 200-500 µg/g + risk factors	Possible mild PEI	Consider ^13^C-MTG or empirical PERT
Normal FE-1 but ongoing malnutrition in pancreatic disease	Functional or post-surgical PEI	Empirical PERT trial (diagnostic–therapeutic)
No pancreatic pathology but low FE-1	Evaluate for secondary/extrapancreatic causes (e.g., DM, IBD, and celiac)	Treat underlying disease + monitor

^13^C-MTG, carbon-13 mixed triglyceride; DM, diabetes mellitus; FE, fecal elastase; IBD, inflammatory bowel disease; PEI, pancreatic exocrine insufficiency; PERT, pancreatic enzyme replacement therapy.

**Table 3.** General Management of PEI: Treatment and Follow-up

**Step/Domain**	**What To Do (Practical Actions)**	**Dosing/Key Thresholds**	**Monitoring and Notes**
Who should be treated?	Initiate therapy in patients with confirmed PEI.	–	Primary aims: improve digestion, symptom burden, and nutritional status; reduce malnutrition-related complications.
Core therapy: PERT	Use PERT as first-line therapy. Individualize dosing; avoid a rigid fixed-dose approach.	Start: 40 000-50 000 lipase units with main meals; 20 000-25 000 with snacks.	Adjust based on symptoms, meal content, and clinical response.
How to take enzymes	Take enzymes with meals; if symptoms persist, consider split dosing across the meal.	Dose to fat load/meal size. Doses above ~120 000 lipase units per meal are rarely required.	Reinforce adherence and correct timing at each visit.
Upper dose safety	Avoid excessive cumulative dosing.	Do not exceed 10 000 lipase units/kg/day (especially emphasized in CF).	High-dose exposure in CF has been associated with fibrosing colonopathy risk.
Enzyme formulation	Prefer preparations containing lipase, amylase, and proteases. Select primarily by lipase content.	–	Standard PERT products are porcine-derived; counsel patients accordingly.
Enteric-coated vs. non-enteric-coated	Enteric-coated products protect enzymes from gastric acid and release in the small bowel. Non-enteric products may require acid suppression (e.g., PPI) in selected cases.	Enteric coatings typically dissolve at pH > 5.5.	Microsphere/minimicrosphere formulations (approximately 1-2 mm) may mix better with chyme.
Nutrition (baseline approach)	Combine PERT with individualized nutritional counseling and adequate calorie intake.	Routine fat restriction is not recommended; optimize enzymes instead.	Small frequent meals may be helpful for some patients; evidence is limited.
Fiber guidance	If symptoms persist despite apparently adequate PERT, consider moderating soluble fiber intake (rather than eliminating fiber entirely).	–	Soluble fiber can reduce enzyme-fat interaction and worsen steatorrhea in some patients.
MCT use	Consider short-term MCT supplementation in selected patients with severe steatorrhea and weight loss.	–	Avoid prolonged MCT-only strategies due to essential fatty acid deficiency risk.
Micronutrients and bone health	Screen and replace deficiencies, including fat-soluble vitamins and key trace elements.	Suggested at diagnosis and at least annually: vitamins A/D/E/K; B12, folate; Mg, Se, Zn, and Fe.	Monitor vitamin D and consider bone density assessment when clinically indicated.
Assessing response	Use combined clinical and laboratory endpoints.	–	Track weight, stool consistency/steatorrhea, abdominal symptoms, and QoL; consider validated symptom tools when available.
Laboratory follow-up	Monitor nutrition-related labs over time.	–	Common options: albumin, prealbumin, and vitamin D (tailor to clinical status).
FE-1 in follow-up	Use fecal elastase mainly for diagnosis/severity stratification rather than routine treatment monitoring.	–	Routine serial fecal elastase measurements are generally not recommended to assess response.
Objective absorption tests (selected cases)	Consider CFA/CNA if objective quantification is required (selected clinical scenarios or research).	–	PERT has been shown to improve absorption indices in studies.
Follow-up interval	Reassess treatment response regularly; shorten intervals if symptoms persist.	Typical reassessment every 3-6 months.	Escalate follow-up for ongoing steatorrhea, weight loss, distension, or malnutrition signs.
If symptoms persist (titration strategy)	Stepwise: confirm adherence and timing; increase dose; add PPI; switch formulation; evaluate alternative causes.	–	Consider bile acid malabsorption or bacterial overgrowth/secondary dysbiosis when response is inadequate.
Adverse effects and safety	Counsel about common mild GI effects; stop therapy; and reassess if hypersensitivity occurs.	–	Possible: distension, nausea, constipation/diarrhea; rare allergic reactions (e.g., urticaria).

CF, cystic fibrosis; CFA, coefficient of fat absorption; CNA, clinical nutrition assessment; FE, fecal elastase; GI, gastrointestinal; MCT, medium-chain triglycerides; PEI, pancreatic exocrine insufficiency; PERT, pancreatic enzyme replacement therapy; PPI, proton pump inhibitor; QoL, quality of life.

**Table 4.** Special Situations in PEI: Practical Treatment Notes

**Special Population/Setting**	**Treatment Approach**	**Dosing/Timing Notes**	**Follow-Up/Key Considerations**
Cystic fibrosis	Apply standard PERT principles with strict attention to weight-based limits.	Total daily dose should remain <= 10 000 lipase units/kg/day.	Dose ceiling is important for safety (fibrosing colonopathy risk with high-dose exposure).
Pancreatic cancer		Typical meal dosing 25 000-50 000 lipase units/meal; consider PPI co-therapy when response is incomplete.	Consider objective steatorrhea definition when available; monitor weight and nutrition closely.
Post-pancreatic surgery	Use standard dosing principles; prioritize prevention of maldigestion and weight loss; tailor to procedure type and symptoms.	Start with standard meal/snack dosing and titrate; consider PPI if response is suboptimal.	Actively screen/correct fat-soluble vitamin deficiencies.
Diabetes (especially type 3c)	Treat PEI and individualize dose dynamically based on symptoms and nutritional course.	Typical meal dosing 25 000-50 000 lipase units/meal	Long-term follow-up may include nutritional labs plus glycemic monitoring (e.g., HbA1c) as clinically appropriate.
Celiac disease	Consider PERT in symptomatic PEI, particularly if symptoms persist despite a gluten-free diet.	Typical meal dosing 25 000-50 000 lipase units/meal Assess response within 1-2 weeks after initiation.	Duration depends on persistence of PEI and overall clinical course.
Inflammatory bowel disease	Use PERT selectively after appropriate evaluation (supportive therapy in selected patients).	Typical meal dosing 25 000-50 000 lipase units/meal; titrate to symptoms.	Track symptoms, weight, stool pattern, and nutritional biomarkers.
Older adults	Start standard dosing and titrate; emphasize nutrition monitoring.	Typical meal dosing 25 000-50 000 lipase units/meal Reassess response within 2-4 weeks after initiation.	Monitor weight, vitamin D, and protein markers closely.
Pregnancy/lactation	Use standard dosing; prioritize maternal nutrition and fetal growth surveillance.	Typical meal dosing 25,000-50,000 lipase units/meal	Monitor symptoms, maternal weight gain, and nutritional status; supplement fat-soluble vitamins when needed.

PEI, pancreatic exocrine insufficiency; PERT, pancreatic enzyme replacement therapy; PPI, proton pump inhibitor.

## Data Availability

The data that support the findings of this study are available on request from the corresponding author.
